# New Developments in Spin Labels for Pulsed Dipolar EPR

**DOI:** 10.3390/molecules191016998

**Published:** 2014-10-23

**Authors:** Alistair J. Fielding, Maria Grazia Concilio, Graham Heaven, Michael A. Hollas

**Affiliations:** School of Chemistry and the Photon Science Institute, The University of Manchester, Oxford Road, Manchester M13 9PL, UK

**Keywords:** electron paramagnetic resonance, spin label, DEER, PELDOR

## Abstract

Spin labelling is a chemical technique that enables the integration of a molecule containing an unpaired electron into another framework for study. Given the need to understand the structure, dynamics, and conformational changes of biomacromolecules, spin labelling provides a relatively non-intrusive technique and has certain advantages over X-ray crystallography; which requires high quality crystals. The technique relies on the design of binding probes that target a functional group, for example, the thiol group of a cysteine residue within a protein. The unpaired electron is typically supplied through a nitroxide radical and sterically shielded to preserve stability. Pulsed electron paramagnetic resonance (EPR) techniques allow small magnetic couplings to be measured (e.g., <50 MHz) providing information on single label probes or the dipolar coupling between multiple labels. In particular, distances between spin labels pairs can be derived which has led to many protein/enzymes and nucleotides being studied. Here, we summarise recent examples of spin labels used for pulse EPR that serve to illustrate the contribution of chemistry to advancing discoveries in this field.

## 1. Introduction

The use of pulsed electron paramagnetic resonance (EPR) to extract magnetic couplings hidden within the linewidth of conventional continuous-wave (CW) studies has led to a resurgence in the applications of EPR [1]. Typically, the couplings are electron-nuclear (hyperfine) or electron-electron interactions, and yield important information about the environment around the unpaired electron. Diamagnetic molecules can be studied by attaching a molecule containing an unpaired electron; known as “spin labelling”. This has led to a significant use in biology through site directed spin labelling (SDSL), which uses mutagenesis to attach labels to specific amino acids. To be able to study the molecular structure of biomacromolecules, techniques such as X-ray diffraction, require crystals from highly purified and monodisperse samples. Likewise, NMR; although able to study polydisperse samples, is limited by the size of the system. EPR in contrast is able to detect the paramagnetic species and ignore diamagnetic background. This makes EPR a very attractive technique for the study of biomacromolecules, possible in membranes and in whole cells.

There is a continuously growing array of pulse sequences [2] used to measure dipolar electron-electron interactions, which have been previously reviewed in detail [3,4]. The dipolar interaction is both distance and orientation dependent, and in principal both parameters can be extracted from pulse measurements. Typically, measurements are made on a frozen solution or in the solid state which causes all orientations of the macromolecule to be observed with respect to the pole faces of the magnet. The most widely applied technique is double electron electron resonance (DEER) or pulsed electron double resonance (PELDOR) which can measure the dipolar interaction between spin pairs and is typically used to extract distances in the range 17–100 Å [5,6,7,8]. In brief, this is a double resonance technique requiring two sets of microwave pulses at different frequencies; an inversion/pump pulse flips one of the spins and inverts the local dipolar field induced by that spin, and an observation echo sequence leads to a refocusing of all other interactions, except the dipolar interaction, which leads to modulation of the echo signal [9]. The overall intensity of the echo signal depends on a characteristic spin relaxation time called the phase memory time (*T*_m_). It is advantageous to maximise *T*_m_ for a longer measurement window needed to measure distances > 40 Å. Longer distances can be measured through deuteration of the protein and buffer due to favourable relaxation [8]. In the case of broad EPR spectra, often found in metals, it is possible to excite specific orientations [10,11] with respect to the magnetic field and obtain structural information [12].

Another powerful technique used to extract the dipolar frequency is called double quantum coherence (DQC) developed by the Freed group [13,14,15]. The DQC method detects a double quantum coherence generated by spin-spin interactions. The methods of DEER and DQC are contrasted in the detailed review of Borbat and Freed [16]. 

Pulsed dipolar EPR has allowed numerous studies on the structure and conformational change of proteins [9] and can harness biological paramagnetic centres such as semiquinones [17], iron sulphur clusters [18] and tyrosyl radicals [19,20]. The techniques have also allowed applications to materials science [21,22]. 

There are many good reviews that focus on how spin labels can provide information about the structure and dynamics of proteins [23,24]. The design of spin labels needs to consider the labels influence on the protein structure as well as EPR spectral properties. The purpose of this article is to describe the general chemical scope of current labels used for pulsed dipolar EPR and to encourage future development.

## 2. Nitroxides

Incorporation of nitroxides by SDSL has, by large, become the dominant technique for DEER distance measurements on proteins [23,24,25]. There is considerable breadth to the understanding of the synthetic chemistry of nitroxides, being by far the most studied and synthesised spin labels [26]. The numerous uses of nitroxide radicals have probably played a part in their success as spin labels. Nitroxides are commonly used as co-oxidants [27], mediators for polymerisation [28], detectors of nitrogen monoxide [29] and for the determination of oxygen concentrations [30]. The considerable stability of nitroxides to organic synthetic conditions, allows their use in organic synthesis, creating an incredible library of spin labels, flexible to the needs of the experimental conditions [26,31,32,33]. 

For pulsed distance measurements, interpretation of EPR data using SDSL requires knowledge of the rotamers and internal dynamics of the respective side chains [24,34]. Different strategies have been used in the past to account for the local dynamics of a spin label in an arbitrary site of a protein. This information is best known for the methanethiosulfonate spin label (MTSL, **1**); also known as R1. Rotamer libraries have been created for α helixes and β sheets [35,36,37], and molecular dynamics (MD) simulations and Monte Carlo conformational searches have been established [38,39], which are commonly used to extrapolate distance measurements between MTSL labels on proteins. There have been several attempts at MD simulations based on DEER constraints [40,41]. 

### 2.1. Nitroxides in the Literature

**Figure 1:**
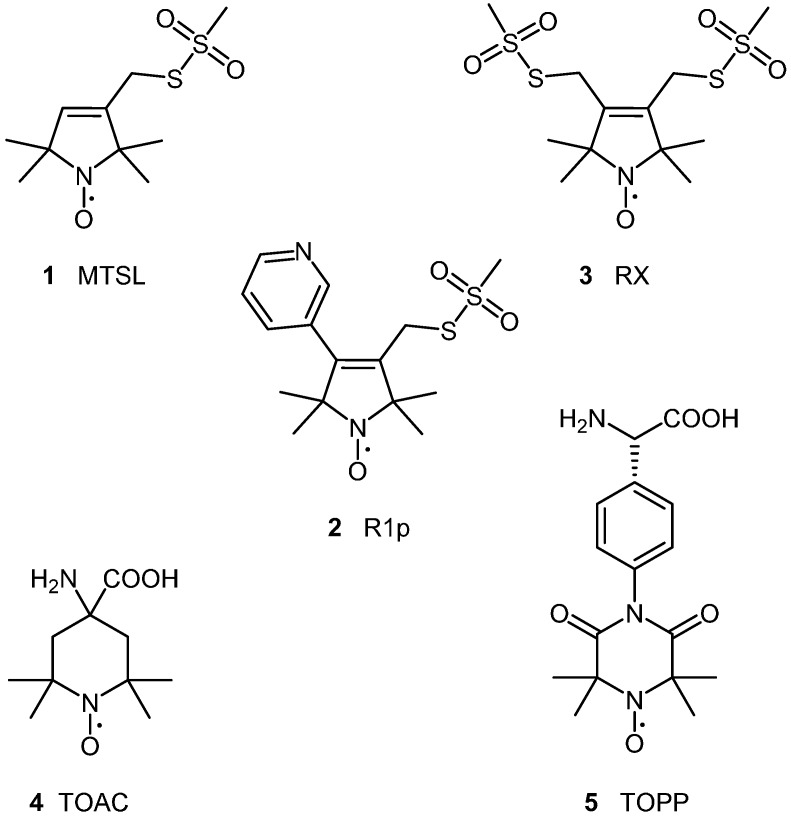
Structures of radical spin labels commonly used for SDSL of proteins.

By far the most popular nitroxide spin label is MTSL (**1**, Figure 1). MTSL is highly selective for thiol groups, and is commonly attached to cysteine residues in proteins. The MTSL side chain (commonly known as R1) also has a minimum impact on the secondary and tertiary structure of proteins. There are, however, disadvantages to R1; the rotational dynamics of the spin label can be complicated and can lead to broad distance distributions being obtained, and both the N-O moiety and disulphide bonds to cysteine residues are susceptible to reducing conditions, such as those that are present inside live cells. Alternative spin labels are available for conditions that are reducing or for in-cell EPR (and will be discussed, in detail, later in this review).

In order to overcome the complexities associated with the flexibility of the R1 cysteine linker, alternative spin label side chains have been created, with significant reductions in the internal motions of the spin label. The 4-pyridyl substituted spin label **2**, gives rise to the R1p side chain, and has been shown to have a considerable reduction in the flexibility of the side chain when in a solvent exposed α-helix [42]. This dramatically simplifies the interpretation of rotamer dynamics, producing narrower distance distributions, leading to greater accuracy. Other spin label side chains with constrained geometry include, RX **3** [43], a bi-functional analogue of R1, which has been shown to tether to two cysteines at positions *i* and *i +* 3 or *i +* 4 on an exposed α-helix. RX can also be positioned at *i* and *i* + 1 in a β strand [44]. 

#### 2.1.1. Spin Labelling of Amino Acids

Non-native amino acids have been incorporated into peptides using solid-phase synthesis using traditional peptide coupling reagents [45,46]. Amino acid spin labels, such as 2,2,6,6-tetramethyl-piperidine-1-oxyl-4-amino-4-carboxylic acid (TOAC, **4**) [46] and 3-amino-1-oxyl-2,2,5,5-tetramethyl pyrrolidine-4-carboxylic acid (POAC) [47] provide a fixed position for a spin label, with no internal rotamers, however, currently can only be incorporated into small peptides and proteins by total synthesis. Incorporation of TOAC via peptide coupling, however, does not occur in good yield [47]; often requiring multiple rounds of peptide coupling, due to the low nucleophilicity of the sterically hindered amino group. TOAC has a very limited range of backbone dihedral angles, creating a significant distortion of the secondary structure of proteins [46]. Despite this, TOAC is commonly used; for example, in studies of the Aib rich peptide alamethicin [48]. In order to combat the distortion of the secondary structure; the 4-(3,3,5,5-tetramethyl-2,6-dioxo-4-oxylpiperazin-1-yl)-l‑phenylglycine (TOPP, **5**) spin label has been introduced, allowing greater flexibility of the backbone chain [49]. 

#### 2.1.2. Spin Labels for DNA and RNA 

**Figure 2:**
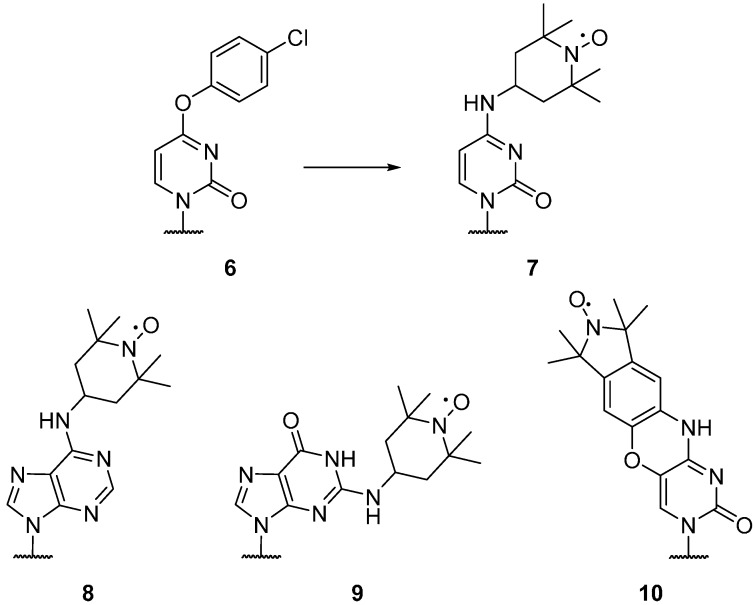
Spin labelled RNA bases.

Nitroxides have been utilised for the specific spin labelling of deoxyribonucleic acid (DNA) and ribonucleic acid (RNA), and can be attached to the bases, phosphate, and sugar backbone of individual nucleotides [50]. DEER measurements on nucleic acids are often carried out using rigid spin labels, which leads to significant orientation selection even at 9 GHz [12]. Commercially available “convertible” nucleotides [51]; *O*^4^-(4-chlorophenyl)-uridine (**6**), *O*^6^-(4-chlorophenyl)-inosine, and 2-fluoroinosine, incorporated into RNA sequences via solid-state synthesis, can be spin labelled using 4-amino-TEMPO; giving the spin labelled RNA bases cytosine (**7**), adenine (**8**) and guanine (**9**), respectively (Figure 2) [52].

**Scheme 1:**
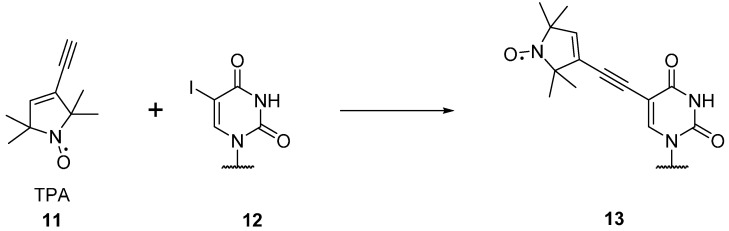
Spin labelling of uracil (**12**).

An alternative rigid, profluorescent spin labelled cytosine nucleotide **10** has been reported by Barhate *et al.* [53], creating a rigid, tricyclic nitroxide labelled base for DNA via a seven step synthesis. This label has also been attached to RNA by Höbartner *et al.* [54] The RNA base uracil can be labelled **13** using alkyne substituted spin labels **11** (Scheme 1), which can be coupled via Sonogashira palladium coupling to 5-iodouridine (**12**) [55,56]. Both five [57] and six [58] membered nitroxideshavebeencoupledto 5-iodouridine, with five membered rings showing greater stability under reducing conditions [59]. The 3-(2-iodoacetamido)-proxyl spin label can be coupled to a 4-thiouridine residue in RNA [60,61]. Nucleotides with alkyne moieties have also been created [62,63], allowing the use of “click chemistry” for post-modification of DNA in solution with azido functionalised nitroxides.

**Scheme 2:**
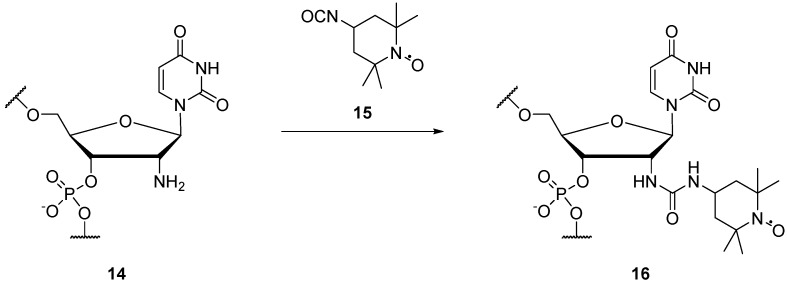
Spin labelling of the 2' position on the sugar backbone of RNA.

The 2' positions on the ribose moiety of RNA have been spin labelled **16** (Scheme 2), from the 2'-amino RNA derivatives **14**, using an isocyanate TEMPO derivative **15** [64,65]. Schiemann *et al.* have demonstrated that this spin label can be used to determine both distance and relative orientation of two spin-labelled nucleosides in DNA at 9 GHz [65].

**Scheme 3:**
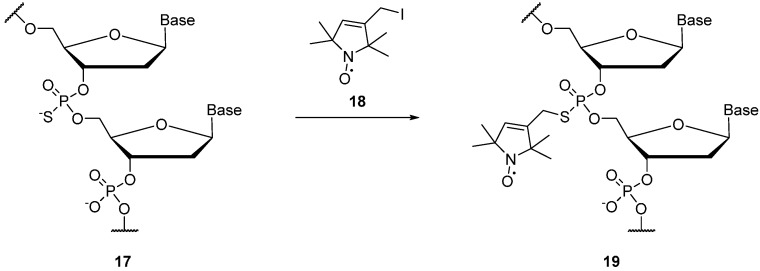
Spin labelling of phosphate backbone of RNA.

The phosphate backbone of RNA/DNA can be spin labelled **19** by reacting the iodo-nitroxide **18** with a commercially available phosphorothioate modified nucleotides **17**, with minimal perturbation of the RNA/DNA complex [66,67] (Scheme 3). Spin labelling of the phosphate backbone is more cost-effective than other nucleotide labelling procedures, as the cost of introducing phosphorothioates is significantly less than incorporating modified nucleotides [67]. 

### 2.2. Design Philosophy 

#### 2.2.1. Parent Ring Structures 

**Figure 3:**
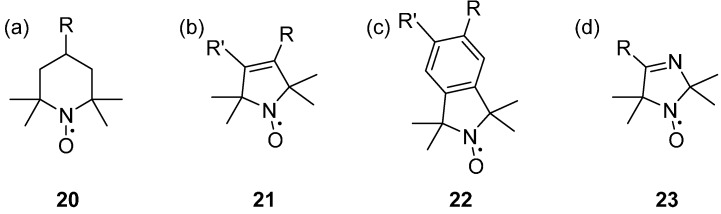
Parent structures of nitroxides; (**a**) a piperidine, (**b**) a pyrroline, (**c**) an isoindoline, (**d**) an imidazoline.

There are three main groups of nitroxides that have been commonly used historically for SDSL; six membered rings (piperidines) **20**, five membered rings (pyrrolines **21** and pyrrolidines), and fused ring systems (isoindolines) **22**; each of which has associated advantages and disadvantages (Figure 3). Nitroxides with a piperidine parent structure are easily derived from triacetoamine [68], with few chemical steps, making cheap and quick functionalised spin labels. Nitroxides with a pyrrole parent structure are synthesised from 4-oxo-TEMPO using a Favorskii rearrangement [26,69,70], requiring additional multi-step synthesis. The six membered piperidine nitroxides are the least stable to chemically reducing conditions, with the five membered pyrroline and pyrrolidine nitroxides having substantially greater resistance [71,72]. 

Fused ring aromatic nitroxides; such as the isoindolines **22**, have been shown to exhibit several advantages over the piperidine and pyrroline nitroxides. Isoindolines have greater structural rigidity, thermal and chemical stability [73], and narrower EPR linewidths [74,75,76] than piperidine nitroxides and can double as fluorescent probes [77], making them useful for in-cell EPR. They can also be highly functionalised, specifically tailoring the spin label to its intended environment [78]; recent advances include the spin labelling of graphene with aryl radical diazonium tetrafluoroborate salts [79]. Imidizolines **23** have been developed to give a largely pH dependent EPR spectrum, and are commonly used as pH probes, and for in-cell pH imaging [80]. 

#### 2.2.2. Alternatives to Methanethiosulfonate Linkages

**Figure 4:**
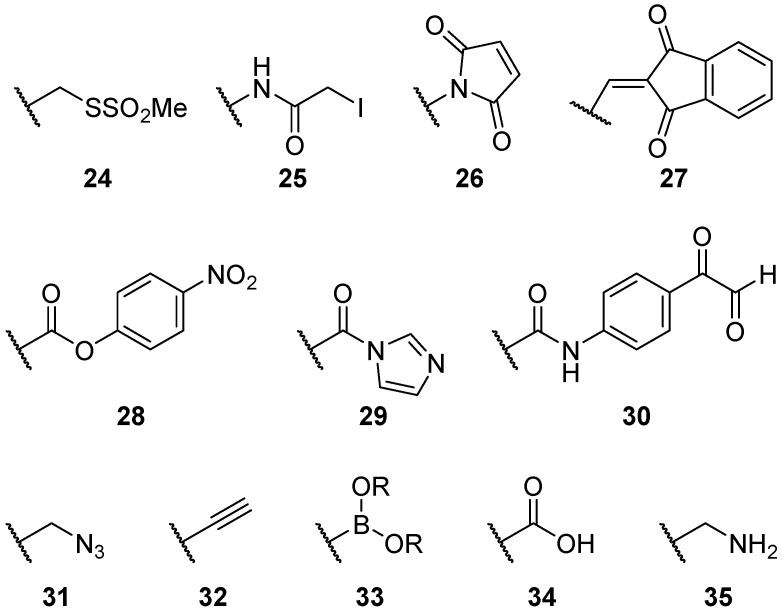
Structures of common nitroxide linkers.

There are many ways of linking nitroxides to proteins and substrates (Figure 4). Methanethiosulfonate moieties **24** are by far the most common used, creating selective disulphide bonds with cysteine residues on proteins. However, there are limitations; it is important to balance the rigidity of a spin label and the distortion of the protein. 

Alternative cysteine specific linkers have also been created, such as the iodocetamido **25** [81] and maleimido **26** [82] and indanedione **27** [83] moieties, which have greater pH stability than the disulphide bonds of the methanethiosulfonate moiety. However, there are unfortunate drawbacks to maleimides; they can react with amines at high pH and they can hydrolyse to the maleamic acid, which may react with other maleimides, reducing their shelf-life [26]. 

Spin labels designed to target different amino acids are also available; with linkers specific to serine (**28**) [84], tyrosine (**29**) [85] and arginine (**30)** [86] reported in the literature. In 2011 an alternative tyrosine linker was developed, utilising a Mannich-type reaction with 4-amino-2,2,5,5-tetramethyl-3-imidazoline-1-yloxy nitroxide [87]. However, the greater scarcity of cysteine residues has meant that the methanethiosulfonate moiety dominates.

“Click chemistry” was developed by Kolb, Finn and Sharpless, as a way to create highly selective, modular carbon-heteroatom bonds, using 1,3-dipolar cycloadditions between organic azides and alkynes in the presence of Cu(I) catalysts [88]. Development of nitroxides containing organic azides **31** and alkynes **32** by Kálai, Hubbell, and Hideg [89], provided a site selective method of incorporating nitroxides into proteins, that was based on chemistry unused by cellular processes. Alkynes **32** may also be coupled to aromatic ring systems via Sonogashira palladium coupling [55,76], and boron esters **33** of pyrrolines [90] have been created, opening opportunities for the creation of increasingly complex radical spin labels.

Carboxylic acids **34** and amines **35** provide simple and reliable linkers for attaching spin labels. Often they are used in combination with each other, creating synthetic amino acids [47,91]. Carboxylic acids can be activated with peptide coupling reagents [92,93], or converted into acyl chlorides [94], facilitating nucleophilic attack via amines. Amine functionality allows the coupling of spin labels to a large array of carbonyls; such as, carboxylic acids, acid chlorides [95] and esters [96]. 

#### 2.2.3. Steric Groups

**Figure 5:**
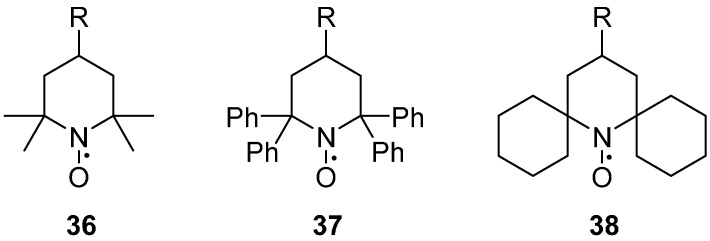
tructures of nitroxides with various steric groups on the 2, 6 positions.

Most nitroxides contain two *gem*-dimethyl substituted quaternary carbons (Figure 5), sterically shielding the nitroxide moiety, and thus providing kinetic stability. This steric stabilisation of the radical, leads to a long lasting, stable nitroxide, but also leads to a decrease of the relaxation time *T*_m_. For standard 2,2,6,6-tetramethyl nitroxides **36**, at temperatures above 70 K, the rotation of the methyl groups is fast enough to average in-equivalent electron-nuclear couplings, dramatically decreasing *T*_m_ [97,98]. When the two *gem*-dimethyl substituted quaternary carbons are replaced either with bulky aromatic groups; such as 1,1,3,3-tetraphenylisoindolin-2-yloxyl (TPHIO, **37**) [99], or with spirocyclohexyl substituents **38** [91,100], the extra source of spin relaxation contributing to *T*_m_ is removed, leading to the low-temperature limit of *T*_m_ already being attained at 80–100 K, while 50–60 K are required for tetramethyl nitroxides. 

**Figure 6:**
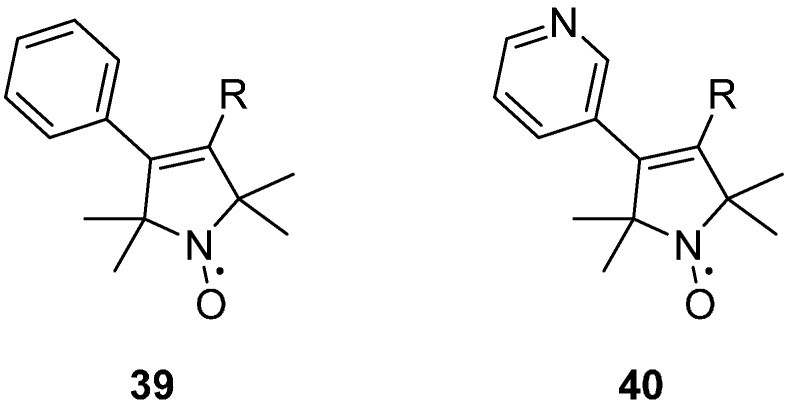
Structures of pyrrolines with rigid substituents on the 4 position.

Steric groups at the 4-position of pyrrolines have been shown to adopt a significantly restricted internal motion than the un-substituted R1 spin label side chain [42]. The presence of a rigid substituent, such as the 4-phenylpyrroline analogue **39** or the 4-pyridylpyrroline **40** (Figure 6), significantly restricts the motion of the side chain at a solvent exposed helical site. The 4‑phenyl substituent is, however, prone to aggregation, most likely due to the increased surface hydrophobicity of the spin label [42].

#### 2.2.4. Isotope Effects

Isotopically labelled analogues of nitroxide spin labels, incorporating N^15^ (*I* = 1/2) into the N‑O moiety, have only two resonances rather than the three for N^14^ (*I* = 1) nitroxides; decreasing spectral width and therefore leading to an increase in signal intensity [101,102]. This is particularly useful to reduce detection limits of dilute systems, most notably for in-cell EPR (see below). Isotope spin labelling has enabled selective distance measurements between N^14^- N^14^ and N^15^- N^15^ spin labels [103]. This is particularly useful for heteropolymers and ensembles of macromolecules; where distance information can become complicated. The ability to distinguish different distance contributions between multiple spin labelled components, significantly decreases the complexity of the spectra. 

## 3. Carbon Centred Spin Labels

### Trityl Radicals 

**Figure 7:**
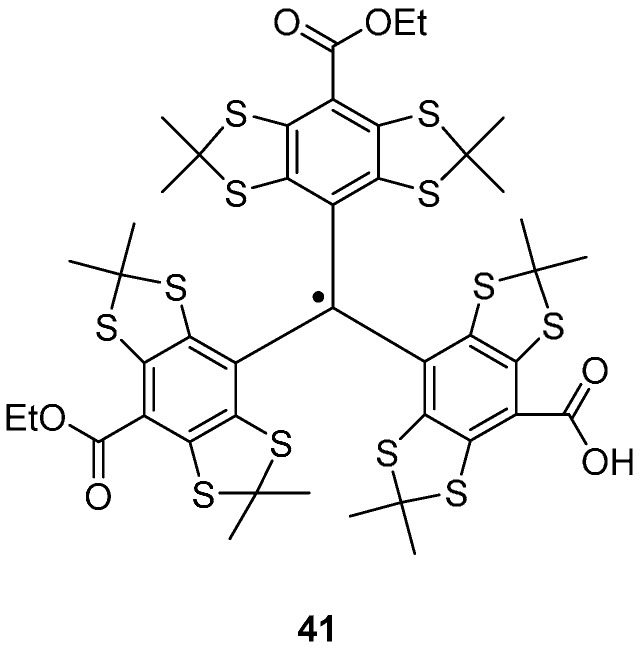
A tetrathiatriarylmethyl radical **41**.

The tetrathiatriarylmethyl (trityl or TAM) radical (Figure 7), has been used as a spin label and offers certain advantages over other spin labels due to its narrow linewidth and relatively long *T_m_* (microseconds at room temperature [104,105]), allowing a more physiological temperature range [106,107,108]. The synthesis of the tetrathiatriarylmethyl radical **41** (Figure 7) is described by Reddy *et al*. [109] and consists of five steps; the first four of which form the trityl alcohol from tetrachlorobenzene, further treatment with boron trifluoride diethyl etherate followed by tin(II) chloride forms the trityl radical. Trityl radicals are stabilised against dimerisation by substituted aryl groups and the in-cell survival time is in the range of hours [110]. 

**Figure 8:**
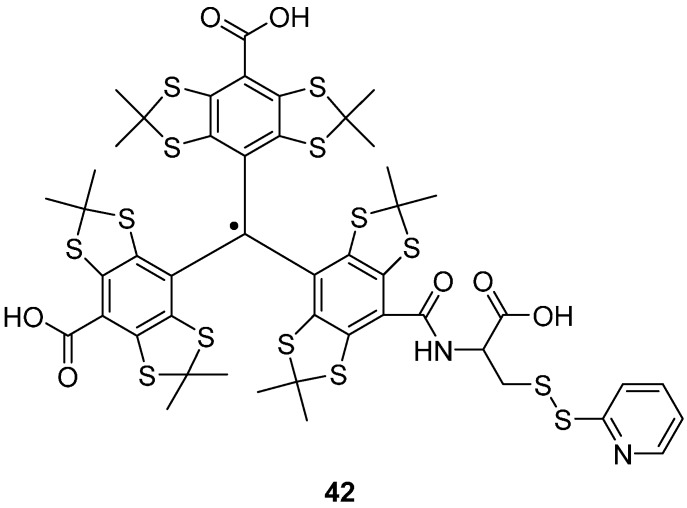
A cysteine specific TAM spin label CT02-TP **42**.

Yang *et al*. have attached TAM labels via disulfide linkages to cysteine residues in T4 lysozyme, using a dithiodipyridine TAM label **42** (Figure 8) [106]. When coupling to cysteine residues on a protein, 2-thiopyridone is also produced, the UV absorbance of which can be used to monitor the reaction [111]. Immobilisation on Sepharose, prevented averaging of the anisotropic dipolar interaction between spins induced by the rotational diffusion of the spin labelled protein. Distance measurements of ~2 nm were successfully performed using DQC in trityl-labelled protein system at 277 K [106].

Further properties of the trityl radical have been investigated by Schiemann group [108,112] who synthesised trityl-trityl and trityl-nitroxide model compounds with well-defined interspin distances in the range of 17 to 49 Å. They studied the nature of the dipolar coupling and observed deep DEER modulations and improved sensitivity in trityl-trityl DQC experiments (of a factor of two) compared to a nitroxide-nitroxide system under the same conditions. The authors note the increased steric bulk compared to nitroxide labels may lead to structural distortions in biological systems, but this will depend on the specific structure, and will (as is the case with nitroxides) have to be checked for structural perturbations caused by the spin label.

**Figure 9:**
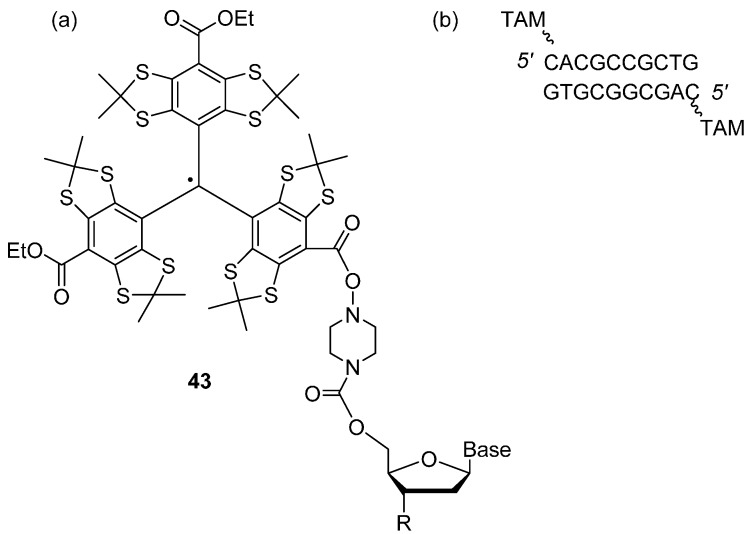
(**a**) TAM-labelled oligonucleotide **43**, (**b**) structure of the doubly-labelled nucleic acid duplex.

Recently, Shevelev *et al.* [107] demonstrated distance measurements of ~4.6 nm in nucleic acid duplex systems at physiological temperatures (310 K) using DQC and at 80 K using DQC and DEER. Distance distributions obtained from DQC measurements at 80 K and at 310 K correlated with the data obtained from DEER measurements at 80 K. The spin labelled nucleic acids, shown in Figure 9a,b, were prepared by coupling the acid chloride TAM spin label to a piperazine activated 5' terminus of two complementary 10-mer oligonucleotides; forming a double stranded spin-labelled nucleic acid duplex, that was subsequently immobilised electrostatically on common ion-exchange sorbent NucleosilDMA.

## 4. Photo-Excited Triplet States

Porphyrin Rings

**Figure 10:**
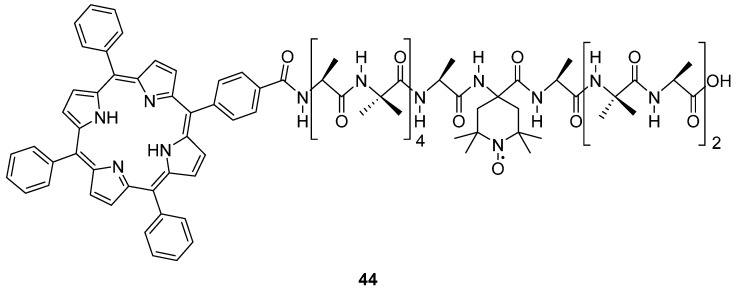
Chemical structure of the bis-labelled peptide TPP-(Ala-Aib)_4_-Ala-TOAC-Ala-(Aib-Ala)_2_-OH **44**.

A recent development in orthogonal labelling is to use a photo-excited triplet state (*S* = 1), localised on a porphyrin moiety coupled to the nitroxide radical (*S* = 1/2) [113]. Among organic chromophores, porphyrins have been widely studied by EPR spectroscopy, due to their high triplet yields, strong spin polarisation, reasonable relaxation rates, and moderate spectral anisotropy caused by the zero-field splitting (ZFS) interaction [114]. The triplet state has distinctive properties compared to metal centres; the large anisotropy due to the ZFS is accompanied by a strong spin polarisation of the spectrum, resulting from a non-Boltzmann population of the triplet-state sublevels by intersystem crossing from the corresponding excited singlet state [113,115]. The strong anisotropy of the triplet state ZFS can be potentially exploited to perform orientation selection; taking advantage of the compensating effect of the spin polarisation signal enhancement. A porphyrin-nitroxide system (bis-labelled peptide TPP-(Ala-Aib)_4_-Ala-TOAC-Ala-(Aib-Ala)_2_-OH) **44** (Figure 10) was synthesised by Di Valentin *et al.* [113] providing proof of the feasibility of using DEER spectroscopy to determine the inter-spin distance. The 15-residue peptide was labelled at the N-terminus with 5-(4-carboxyphenyl)-10,15,20-triphenylporphyrin (TPP) and at position 10 with TOAC. The peptide bridge connecting the paramagnetic probes consists of alternating L-alanine (Ala) and α‑aminoisobutyric acid (Aib) residues; known to promote an α-helix conformation and consequently a well-defined geometry in terms of distance, relative orientation, and restricted conformational flexibility. 

The DEER spectrum revealed a well-resolved and pronounced dipolar modulation. The significant extent of orientation selection expected in the relatively broad porphyrin triplet spectrum was not visible on the DEER spectrum and attributed to an observer position exciting almost all the possible molecular orientations with the tetrapyrrole plane parallel to the magnetic field, the lack of co-linearity between the zero field splitting principal axes and the spin−spin distance vector, and to a lesser extent by the small degree of rotational freedom of the para-substituted benzoyl group on the porphyrin. It is envisaged that this type of orthogonal approach can be extended to chromophores whose triplet state is highly polarised and well-characterised by EPR spectroscopy, such as porphyrin derivatives, fullerenes, and flavins [113,114,115]. It is also thought that chlorophylls and flavins could be exploited as endogenous photo-probes because of their presence in several classes of proteins.

## 5. Transition Metals 

Many efforts have been recently devoted to the development of alternative spin labels with more attractive properties than conventional nitroxide radicals. Metals, such as copper (II), gadolinium (III), manganese (II) and nickel (II) have been utilised [116]. These systems present several EPR features, differing from nitroxide spin labels; firstly, they can have large hyperfine splitting and *g*-tensor anisotropy, which results in broad EPR spectra of which microwave pulses may only excite a fraction, resulting in strong orientation selection [117]. Secondly, relaxation rates are normally fast and limit the detection window and the accessible distance range. And thirdly, spin density can be distributed into the ligands, leading to the breakdown of the simple point-dipole model and the onset of an exchange coupling *J* [118]. 

### 5.1. Gadolinium (III)

Gadolinium (III) complexes have been utilised as spin labels for distance measurements in rigid and flexible model systems [119], for proteins [120,121,122] and for oligonucleotides [123], and are summarised in a recent review by Goldfarb [124]. 

There are several advantages in using Gd(III) ions (*S* = 7/2); firstly, the narrowing of the spin transition from m_s_ = −1/2 to m_s_ = 1/2 leads to increased sensitivity when using high magnetic fields. Secondly, the high transition probability of the *S* = 7/2 gadolinium spin with respect to the *S* = 1/2 nitroxide spin allows shorter microwave pump pulses for an equivalent microwave magnetic field. Lastly, the short spin-lattice relaxation time of Gd(III) complex allows the use of shorter repetition times, and the isotropic excitation resulting from the broadly distributed strengths and orientations of the ZFS typically suppresses orientation selection effects [21]. Gadolinium DEER measurements are usually performed at 25 K at a single magnetic field position, neglecting the orientation. 

**Figure 11:**
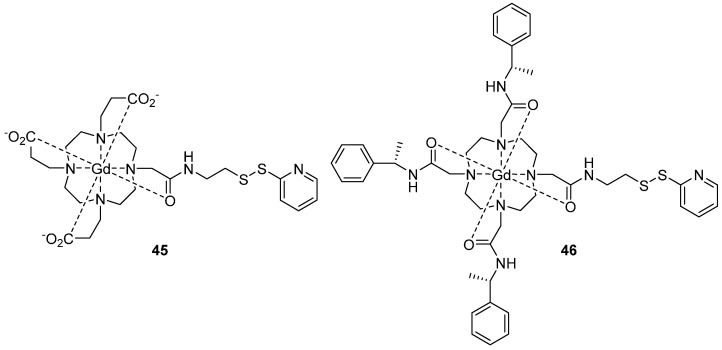
Gadolinium (III) spin labels DOTA chelate **45** and C1 **46** with phenylethylamine substituents.

These advantages were demonstrated by Yagi *et al.* using Gd(III)-DOTA spin label **45** through reaction with a cysteine thiol groups to measure with outstanding accuracy distances of 6 nm between Gd(III) pairs at 95 GHz [122]. Furthermore, Matalon *et al.* [125] used gadolinium spin labels (Figure 11) to study transmembrane helical WALP peptides (composed of tryptophan (W), alanine (A) and leucine (L) amino acids) in model 1,2-dioleoyl-sn-glycero-3 phosphocholine (DOPC) vesicles systems. AWALP23 peptide was labelled at N and C termini cysteine residues with two different Gd-DOTA derivatives, DOTA **45** and C1 **46**. DEER measurements were performed at 95 GHz, showing distances of 3.7 nm and 4.3 nm with WALP23-DOTA 45 and WALP23-C1 46, respectively. The synthesis of C1 is described by Graham *et al*. [126].

**Figure 12:**
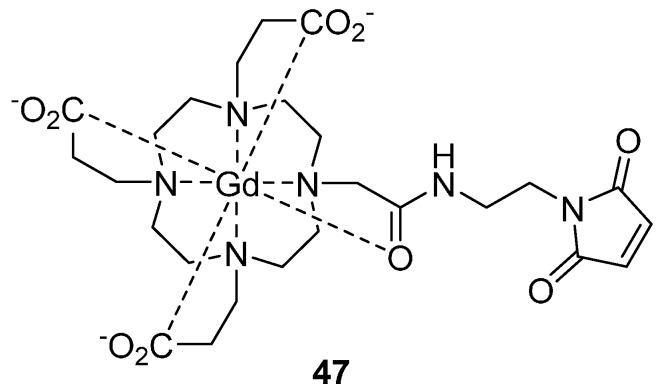
The Gd(III) spin label **47**; chelated by maleimido mono amide 1,4,7,10-tetraazacyclododecane-1,4,7,10-tetraacetic acid (DOTA), which can attach to the thiol group of cysteine residues.

Gd(III) centres have also been used in combination with nitroxide radicals [127,128,129]. In particular, a combination of selective distance measurements in nitroxide-nitroxide, Gd-nitroxide and Gd-Gd pairs is of potential interest for structural studies of macromolecules and other nano-objects. In the Gd-nitroxide orthogonal spin pair; each type of paramagnetic centre opens the possibility to characterise the local environment of each spin probe, and macromolecular aggregation, without the need of additional singly labelled samples. This approach demonstrates good performance in distance measurements, and possessed high sensitivity using high-field EPR in protein systems [127,128,130,131]. Garbuio *et al*. [127] have used the Gd‑nitroxide orthogonal approach; to study bacteriophage T4-lysozyme using MTSL and the Gd(III) spin label **47** (Figure 12).

### 5.2. Copper (II)/Nickel (II) Porphyrin/Nitroxide Systems 

Copper (II) centres often occur in biological systems and play an important role in the macromolecular architecture as well as the structure and function of proteins and oligonucleotides. At 9 GHz the Cu(II) EPR spectrum extends over 700 G due to its *g‑*anisotropy and hyperfine coupling (*I* = 3/2 nucleus, ^63^Cu and ^65^Cu isotopes) so that the microwave pulses used in DEER typically excite only a small part of the spectrum, usually less than 50 MHz [12]. In DEER studies on Cu-nitroxide and Cu-Cu model systems [132,133], excitation in the *g_⊥_* region of the copper spectrum led to negligible orientation selection and allowed a reasonable analysis of a distance distribution. 

In order to resolve orientation effects, high quality experimental DEER data is required due to the small modulation depths and subtle changes with orientation [12]. Yang *et al.* have studied the optimal EPR conditions for data acquisition in the case of Cu-nitroxide and Cu-Cu systems with regard to commercial instrumentation [134]. Bode *et al.* have studied the influence of orientation selection, spin-density distribution, conformational flexibility, and exchange coupling on DEER measurements at 9 GHz for two structurally related nitroxide-labelled Cu(II) and Ni(II) porphyrin systems [118]. Cu(II)-Cu(II) distances have also been measured in proteins [116], such as the homotrimeric Cu-containing nitrite reductase [135], and the DNA modifying enzyme EcoRI endonuclease homodimer bound to its specific DNA recognition site [136].

## 6. Expression in Cells

### 6.1. Introducing Spin Labels via Unnatural Amino Acids 

The complexity of measuring multiple spin pair interactions in proteins is usually avoided through mutations so that a single cysteine pair can be introduced in the desired positions. In the case of larger proteins when there may be a large number of cysteine residues, several rounds of mutagenesis will be required which is both time consuming and has an increased chance of perturbing the important structural and functional role played by these residues. Therefore, alternative labelling strategies are required to increase the scope of SDSL distance measurements to more complex problems. 

One approach for spin labelling proteins is to use endogenous expression of unnatural amino acids [137,138]. These can be coded into the DNA by an amber stop codon (three letter code TAG) allowing the unnatural amino acids to be inserted into the sequence or to substitute a native residue, leaving all other amino acid residues untouched. This method works by introducing a suppressor tRNA which recognises amber stop codons in DNA and introduces the unnatural amino acid for coupling to the growing peptide. This technique is technically challenging because as well as synthesising an unnatural amino acid, a mutated tRNA-aminoacyl synthetase enzyme has to be evolved which can catalyse the esterification of the unnatural amino acid to the suppressor tRNA. Nonetheless over thirty different unnatural amino acids have been incorporated into proteins this way [138].

For introducing spin labels to proteins, this method has been used in two different ways: by introducing amino acids with paramagnetic side chains; or, by introducing unnatural amino acids with side chains of orthogonal chemical reactivity to the twenty natural amino acids, allowing subsequent spin labelling.

#### 6.1.1. Unnatural Amino Acids with Paramagnetic Centres

As well as being able to retain native cysteines, expressing unnatural amino acids with paramagnetic centres has the advantage over other spin labels that the conformational degrees of freedom of the side chain are limited, as with short spin labelled peptides produced by solid phase synthesis. Since the intracellular environment is strongly reducing, the main synthetic challenge is to design an amino acid with a paramagnetic centre which can persist in cells without being reduced to the corresponding hydroxylamine over the several hours necessary for protein expression.

**Figure 13:**
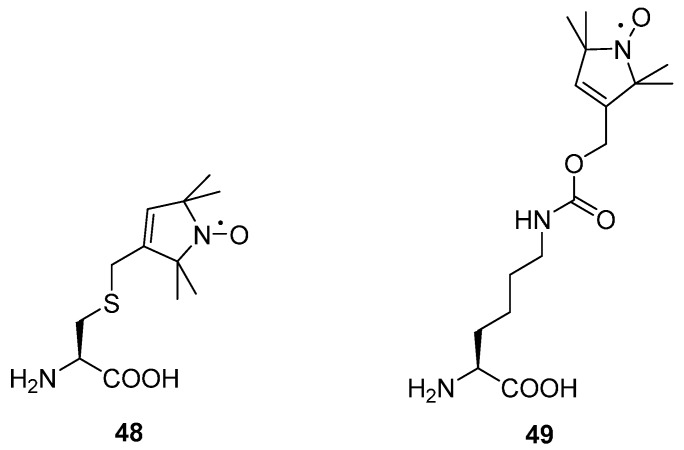
Paramagnetic unnatural amino acids **48** and **49**.

The first example of an endogenously expressed paramagnetic amino acid was demonstrated in 1994 using T4 lysozyme, singly labelled with the nitroxide linked amino acid **48** (Figure 13). CW EPR was used to measure the label in the purified protein [139]. Distance measurements require at least two labels but introducing more than one unnatural amino acid requires having multiple amber stop codons in the DNA sequence which can be recognised by the cellular machinery for termination of the growing peptide, leading to lower yields of expression. Recent developments in molecular biology have allowed proteins to be expressed in *E. coli* where this mechanism has been removed, rapidly expanding the applicability of this method [140,141,142,143]. The first example of DEER on protein with unnatural paramagnetic amino acids was reported earlier this year with the unnatural nitroxide amino acid **49** [144].

#### 6.1.2. Unnatural Amino Acids with Orthogonal Chemical Reactivity

Introducing unnatural amino acids with orthogonal reactivity to the natural twenty amino acids allows selective labelling after expression. This allows a much greater scope for labelling strategies, however, the side chains are generally longer than when the paramagnetic centre is endogenously expressed.

**Figure 14:**
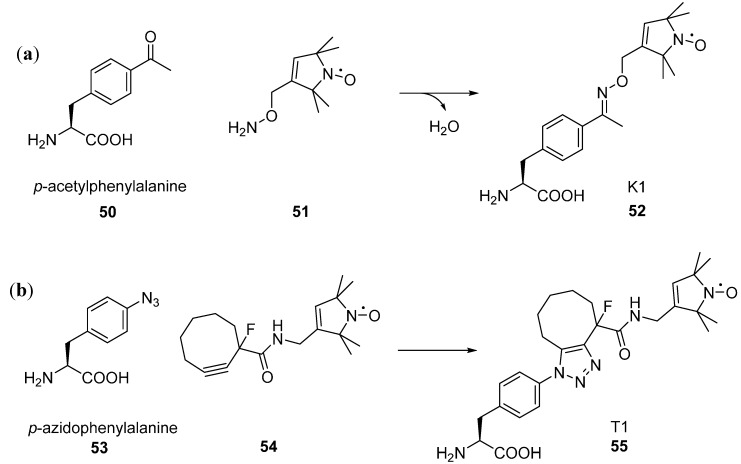
Spin labelling of unnatural amino acids (**a**) *p*-acetyl phenylalanine **52** and (**b**) *p*-azidophenylalanine **55**.

The unnatural amino acid *p*-acetyl phenylalanine **50** [145] contains a ketone group which can be reacted with the hydroxylamine **51** to give the ketoxime linked nitroxide side chain K1 **52** (Figure 14) and distances have been determined by DEER [146]. However, this reaction was demonstrated at pH 4 and required several hours at 37 °C; conditions which would not be tolerated by many proteins.

Click chemistry offers an opportunity for pH neutral labelling, although the use of Cu(I) as a catalyst is undesirable due to cytotoxicity and the need to maintain reducing conditions unfavourable with nitroxides. Copper-free click chemistry has been used for fluorescent labelling of unnatural amino acids [147] and subsequently for spin labelling [148]. The unnatural amino acid *p*-acetyl phenylalanine **53** with a cyclo-octyne label **54** gives a triazole linked nitroxide side chain **55** [148] (also referred to as T1 [24] in a metal-free synthesis. However, dipolar EPR distance measurements have not yet been published using a pair of these spin labels.

## 7. In-Cell EPR

Unnatural amino acid mutagenesis allows opportunities for detection of EPR signals in-cell without purification of the protein and continuous-wave EPR measurements have been recorded using unnatural amino acid **49** [144]. But the concentration of proteins expressed is limited and no in-cell dipolar EPR measurements have currently been reported using this method.

In-cell EPR has largely been approached by the microinjection of spin labelled samples into cells; using oocytes of the African frog *Xenopus laevis*. A potential problem was the reducing cellular environment; which could convert a nitroxide into its corresponding hydroxylamine. Therefore, methods involving microinjection require freeze-quenching the sample after a short incubation period.

**Figure 15:**
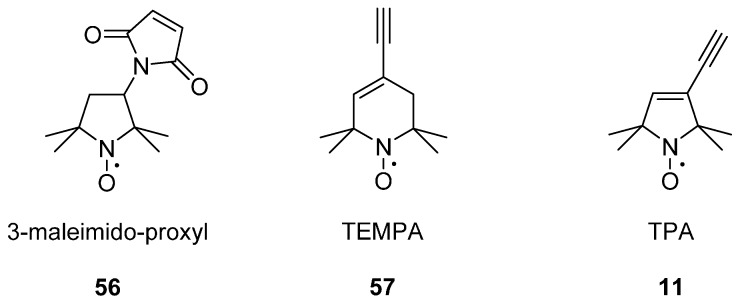
Spin labels used for in-cell DEER. The cysteine binding 3-maleimido-proxyl **9**, and nucleic acid base binding labels TEMPA **57** and TPA **11**.

The first in-cell DEER was reported in 2010 on the protein ubiquitin labelled with 3-maleimido-proxyl **56** (Figure 15), microinjected into *Xenopus laevis*, then freeze-quenched to prevent nitroxide reduction [149]. The maleimido linker group was chosen for its resistance to reductive cleavage.

In-cell DEER has also been carried out on nucleic acids using the spin labels TEMPA **57** [58] and TPA **11** [57,150] (Figure 15). It has been observed that TEMPA **57** injected cells had to be freeze‑quenched in as little as ten seconds [58], whereas cells containing TPA **11** linked nucleic acids could be incubated for up to seventy minutes because 5-membered ring nitroxides are significantly more stable than 6-membered ring nitroxides to reducing cellular conditions [59,71,72]. These longer incubation times of TPA **11** were preferable as this allowed nucleic acid conformations to reach equilibrium in the intracellular environment [150].

### In Situ His Tag Labelling

**Figure 16:**
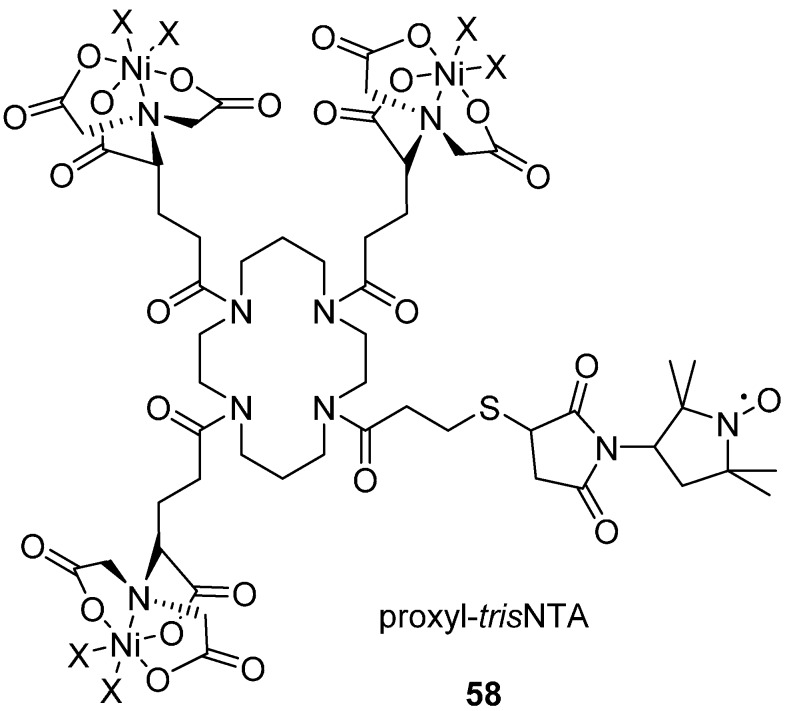
Histidine tag spin label proxyl-*tris*NTA **58**. X = histidine coordination site.

Poly-histidine tags of recombinant proteins have been labelled *in situ* in the presence of nickel using a proxyl-*tris*NTA label **58** (Figure 16), allowing selective labelling from crude cell lysates and EPR measurement without purification [151].

The proxyl-*tris*NTA spin label **58** contains a proxyl nitroxide reporter group along with three nitrilotriacetic acid (NTA) groups which exploit the binding affinity of polyhistidine tags for nickel loaded NTA, which is commonly used as a protein purification strategy for polyhistidine tagged proteins. The short *T*_m_ relaxation time of the nickel bound label meant that it was not practical to measure the distance between a pair of these labels by DEER; but this label was usefully demonstrated as a pump spin with a separate MTSL as an observer spin.

## 8. Conclusions

Spin labelling techniques combined with pulse EPR techniques have allowed greater insight into biological structures particularly enriching our conformational mobile view of many proteins. The most widely used label is MTSL comprising a nitroxyl radical attached to a five-membered ring with a methanethiosulfonate moiety. In the case of nucleotides, TOAC has readily allowed the determination of orientation as well as distance. Gd(III) and trityl labels have also been validated for accurate dipolar measurements. Importantly, orthogonal spin labelling strategies have permitted discriminatory structural information. The need to measure inside live cells has resulted in promising experiments using nitroxides and this area is of active research. The use of Gd(III) and trityl labels have great potential for measurements within cells because of their favourable reduction properties. The novel idea to use photo-EPR sensitive labels has potential under well-chosen conditions. The advent of methods to incorporate unnatural amino acids into proteins which have multitudes of cysteine residues allows rich future biological targets. It is envisaged that further advances incorporating unnatural amino acids combined with novel labels, as well as developments in instrumentation [152], will lead to increased growth in EPR as a biochemical tool. Further applications of dipolar spectroscopy using labels to study materials are on-going in many laboratories. The design of future labels needs to consider EPR spectral features; resonance width, spin delocalisation and intensity, as well as chemical factors; reduction potential and molecular size. Greater rigidity of labels is also advantageous to allow orientation determination.
